# Supplementary material for: Dynamic CYP2E1 expression and metabolic activity changes in male rats during immune liver injury and sex differences in alcohol metabolism
Source: PLoS One. 2025 Jun 18;20(6):e0325135. doi: 10.1371/journal.pone.0325135 (PMC12176122; doi:10.1371/journal.pone.0325135)
Supplement: Attachment — Submitted filename: Response_to_Reviewers_auresp_2.pdf [file pone.0325135.s003.pdf]

6. Review Comments to the Author

Please use the space provided to explain your answers to the questions above. You may also include additional comments for the author, including concerns about dual publication, research ethics, or publication ethics. (Please upload your review as an attachment if it exceeds 20,000 characters)

Reviewer #1: The manuscript has improved significantly; the errors have been corrected and the results clarified. Moreover, the authors have properly addressed and discussed each of the comments raised during the first round of review. For the work to be suitable for publication, I have a few comments regarding the figures.

-As requested, the panel corresponding to β-tubulin (Figure 5B) has been replaced. Please ensure that the panel uses the same format and style for the border/frame in order to maintain consistency and visual coherence across the figure. Also related to the Western blots, it appears that the bands identified as p38 (41 kDa) in the main figures may not correspond to the marked ones on the uncropped membranes shown in the Supporting Material. I consider it essential for publication that this point be carefully reviewed. I wonder which bands were used for the quantification, especially considering that the intensity pattern of the bands indicated on the uncropped membranes appears to differ from those shown in the paper. For example, for p38, the pattern does not seem to vary between the control and 6d groups in the main figure, while in the uncropped membranes, the control lanes appear notably fainter than those of the 6d group.

-Figure 4: In this version, the authors have modified the graph (4C) in response to the first review request; however, the colors used in the bars do not match those in graph B, which causes confusion. Graph D should also follow the same logic.

-Figure 5: Graphs G and H do not follow the same color reference used in the previous figures. Please edit the figures to ensure consistency and make them self-explanatory.

Additionally, I believe one im_portant point raised by the authors in their response should be explicitly mentioned in the Materials and Methods section. Specifically, authors stated:

"Reply: Fig. 4 and Fig. 2 are two different experiments, using two batches of rats. The metabolic activity of CYP2E1 varies greatly and is influenced by many factors, such as diet and environment. Even for a group of mice in the same experiment, the data dispersion is relatively large. To reduce errors, we adopted the method of taking one from each group for simultaneous measurement to minimize the influence of the environment on the results."

I consider it im_portant to include this clarification in the Materials and Methods section so that readers do not have the same doubt and to further strengthen the interpretation of the presented results.

Reply:

1.The manuscript has been revised to ensure consistency between the p38 band (41 kDa) in Figure 5 of the paper and the corresponding band marked on the uncut film presented in the supporting information. The p38 strip in the paper is accurate; however, the position of the box in the supporting material was incorrect and has now been corrected.

To maintain alignment between the supporting materials and the figures in the manuscript, the relevant images in the paper have also been updated.

2. Figures 4(C, D) have been adjusted and revised to harmonize the color schemes used in the bar chart with those in Figure B, thereby minimizing potential confusion.

3. Figure 5 has been revised to ensure that the colors in Figures G and H match those in the preceding figure.

4. This clarification has already been incorporated into the Materials and Methods section. It should be noted that Figures 4 and 2 represent two distinct experiments conducted using two separate batches of rats.
